# The Pediatric Microbiota–Gut–Brain Axis: Implications for Neuropsychiatric Development and Intervention

**DOI:** 10.3390/children12111561

**Published:** 2025-11-17

**Authors:** Giuseppe Marano, Greta Sfratta, Ester Maria Marzo, Giorgia Cozzo, Francesca Abate, Gianandrea Traversi, Osvaldo Mazza, Esmeralda Capristo, Eleonora Gaetani, Marianna Mazza

**Affiliations:** 1 Unit of Psychiatry, Fondazione Policlinico Universitario A. Gemelli IRCCS, Largo Agostino Gemelli 8, 00168 Rome, Italy; 2 Department of Neurosciences, Università Cattolica del Sacro Cuore, 00168 Rome, Italy; 3 Pediatric Unit, Fondazione Policlinico A. Gemelli IRCCS, 00168, Rome, Italy; 4 Unit of Medical Genetics, Department of Laboratory Medicine, Ospedale Isola Tiberina-Gemelli Isola, 00186 Rome, Italy; gianandrea.traversi@gmail.com; 5 Spine Surgery Department, Bambino Gesù Children’s Hospital IRCCS, 00168 Rome, Italy; osvaldo.mazza1973@hotmail.it; 6 Department of Translational Medicine and Surgery, Fondazione Policlinico Universitario A. Gemelli IRCCS Università Cattolica del Sacro Cuore, 00168 Rome, Italy; 7 Department of Medical and Surgical Sciences, Fondazione Policlinico Universitario A. Gemelli IRCSS, 00168 Rome, Italy; 8 Unit of Internal Medicine, Cristo Re Hospital, 00167 Rome, Italy

**Keywords:** pediatric microbiota, gut–brain axis, neurodevelopment, neuropsychiatric disorders, autism spectrum disorder (ASD), attention-deficit/hyperactivity disorder (ADHD), psychobiotics, early-life interventions

## Abstract

**Highlights:**

**What are the main findings?**

- Alterations in the pediatric gut microbiota are linked to neurodevelopmental and psychiatric conditions such as ASD, ADHD, and mood disorders.
- Mechanistic pathways include immune signaling, microbial metabolites, and neural communication via the vagus nerve and enteric nervous system.

**What is the implication of the main finding?**

- Early-life modulation of the gut microbiota may reduce the risk or severity of neuropsychiatric disorders in children.
- Interventions such as probiotics, diet, and psychobiotics show promise but require more pediatric-specific clinical research.

**Abstract:**

Background: The gut microbiota plays a crucial role in brain development and function, especially in early life. Disruptions in the pediatric microbiota–gut–brain axis have been linked to neurodevelopmental and psychiatric disorders. We hypothesize that early-life dysbiosis can perturb neurodevelopment via the pediatric microbiota–gut–brain axis, increasing risk and/or severity of neuropsychiatric outcomes, and that microbiota-targeted strategies may mitigate this risk. Methods: We conducted a narrative review by searching PubMed, Scopus, and Web of Science up to January 2025 for studies addressing pediatric microbiota, neuropsychiatric development, and interventions. Human and animal studies were included if they provided mechanistic or clinical insights. Results: Key determinants of microbiota development in childhood include mode of delivery, feeding practices, antibiotic exposure, diet, and environment. Altered microbial composition has been associated with autism spectrum disorder, attention-deficit/hyperactivity disorder, mood disorders, anxiety, and anorexia nervosa. Mechanistic pathways involve immune modulation, neural signaling (including the vagus nerve and enteric nervous system), and microbial metabolites such as short-chain fatty acids. Interventions targeting the microbiota—ranging from dietary strategies and probiotics to psychobiotics and fecal microbiota transplantation—show promise but require further pediatric-focused trials. Conclusions: The pediatric microbiota–gut–brain axis represents a critical window for neuropsychiatric vulnerability and intervention. Early-life strategies to support a healthy microbiota may help reduce the risk or severity of psychiatric disorders. Future research should prioritize longitudinal pediatric cohorts and clinical trials to translate mechanistic insights into precision interventions.

## 1. Introduction

**Figure 1:**
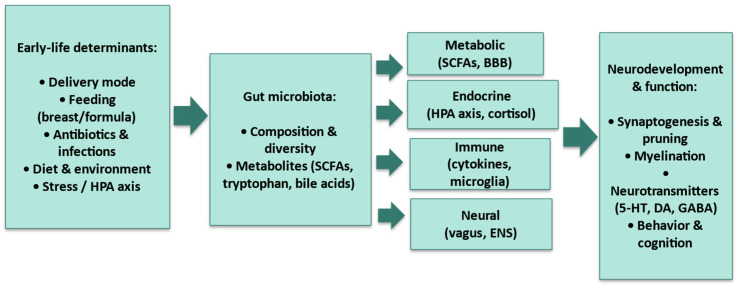
Pediatric microbiota–gut–brain axis.

The human gastrointestinal microbiota, composed of trillions of microorganisms, is essential for physiological homeostasis. In childhood, its composition and diversity undergo rapid changes influenced by factors such as delivery mode, diet (e.g., breastfeeding vs. formula), antibiotic exposure, and environmental interactions [1]. Beyond immune system maturation, the gut microbiota impacts brain development and function via the microbiota–gut–brain axis [2]. In pediatric populations, a diverse and balanced gut microbiota is vital for healthy neurological development. Early-life dysbiosis, or disruption of microbial colonization, has been linked to increased risk of neurodevelopmental and neuropsychiatric disorders, including autism spectrum disorder (ASD), attention-deficit/hyperactivity disorder (ADHD), and anxiety [3]. Evidence increasingly supports a bidirectional communication between the gut and the central nervous system (CNS), mediated by neural (e.g., vagus nerve), endocrine (e.g., cortisol), immune, and metabolic (e.g., Short-chain fatty acids-SCFAs, tryptophan metabolites) pathways [4]. Alterations in these pathways during critical periods may influence cognitive, emotional, and behavioral development. As outlined in Figure 1, the pediatric microbiota–gut–brain axis integrates early-life determinants with gut microbial features and four major signaling routes, neural, immune, endocrine, and metabolic, that converge on synaptogenesis/pruning, myelination, neurotransmission, and behavior.

Schematic overview of the pediatric MGBA. Early-life determinants (delivery mode, feeding, antibiotics/infections, diet/environment, stress/HPA activation) shape gut microbiota composition, diversity, and metabolites (e.g., short-chain fatty acids, tryptophan, bile acids). Communication occurs via four principal routes- neural (vagus, ENS), immune (cytokines, microglia), endocrine (HPA axis, cortisol), and metabolic (SCFAs, BBB integrity), influencing neurodevelopmental processes including synaptogenesis/pruning, myelination, neurotransmitters (5-HT, DA, GABA), and behavior/cognition. Abbreviations. SCFAs: Short-Chain Fatty Acids; ENS: Enteric Nervous System; HPA: Hypothalamic–Pituitary–Adrenal (axis); BBB: Blood–Brain Barrier; 5-HT: Serotonin; DA: Dopamine; GABA: γ-Aminobutyric Acid.

This review aims to explore the role of microbiota in pediatric neuropsychiatric conditions, with potential clinical implications including therapeutic strategies using probiotics, prebiotics, and dietary interventions to modulate the intestinal ecosystem in at-risk populations. Several environmental and biological factors shape the establishment of the gut microbiota during early life. Early-life alterations in the gut microbiota (dysbiosis) can influence neurodevelopment via the microbiota–gut–brain axis. Accordingly, our working hypothesis is that perturbations to early-life gut ecosystems causally contribute to adverse neurodevelopmental trajectories via neural, immune, endocrine, and metabolic signaling, and that targeted interventions may confer benefit during pediatric sensitive periods.

Table 1 summarizes the main determinants, highlighting their potential implications for microbiota development and, consequently, neuropsychiatric health.

## 2. Methods

This narrative review was conducted by searching PubMed, Scopus, and Web of Science up to January 2025 using combinations of the keywords “pediatric,” “gut microbiota,” “microbiota-gut–brain axis,” “neurodevelopment,” and “neuropsychiatric disorders.” Studies were included if they focused on pediatric populations (0–18 years) and explored associations between gut microbiota and neuropsychiatric outcomes or interventions. Both human and relevant preclinical studies were considered. Exclusion criteria included adult-only studies, non-English publications, and papers without relevance to neuropsychiatric development. Reference lists of eligible studies were hand-searched to identify additional sources. Evidence was synthesized narratively, with emphasis on developmental trajectories, disorder-specific microbiota alterations, and potential interventions. A total of 612 records were identified through database searches (PubMed, Scopus, and Web of Science). After removing 209 duplicates, 403 titles and abstracts were screened for relevance to early-life gut microbiota and neurodevelopmental or psychiatric outcomes. Of these, 278 articles were assessed in full text, and 237 studies met the inclusion criteria and were incorporated into the final narrative synthesis.

**Figure 2:**
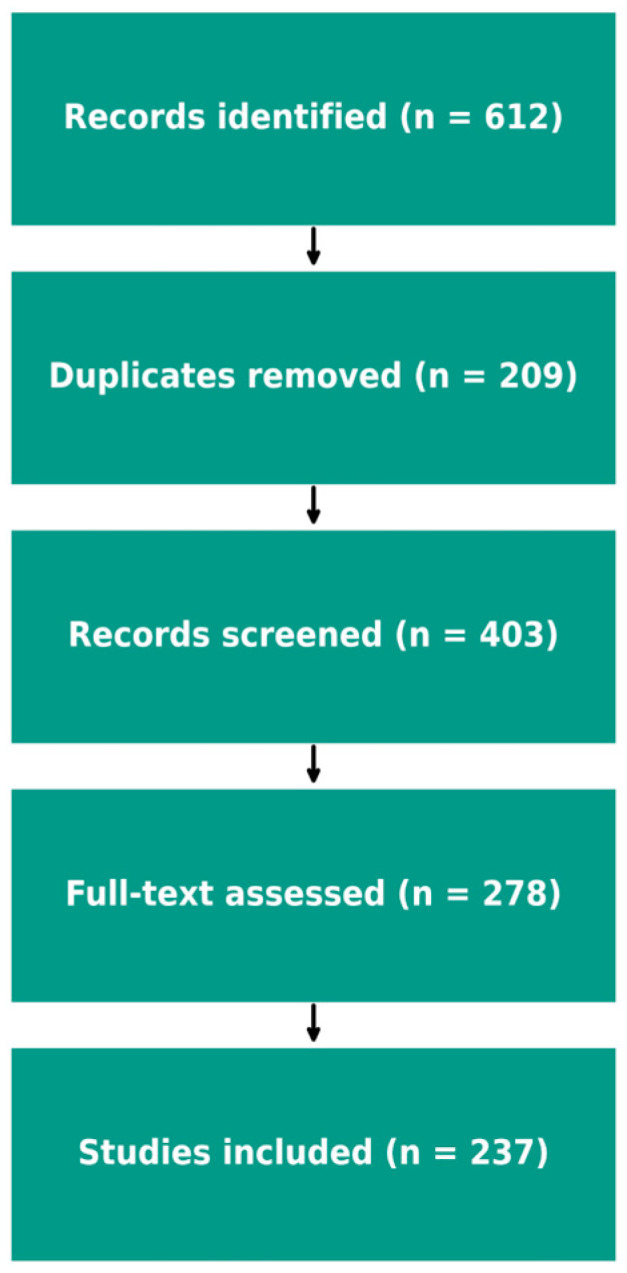
PRISMA flow diagram illustrating study identification and selection.

The selection process is illustrated in the PRISMA-style flow diagram (Figure 2) to enhance transparency regarding study identification, screening, eligibility, and inclusion.

## 3. Early-Life Development of the Gut Microbiota

### 3.1. Neonatal Colonization: Vaginal Delivery vs. Cesarean Section

The gut microbiome is established at birth and rapidly matures, reaching an adult-like composition by 12–36 months [5]. Microbiota–gut–brain interactions begin in utero, influenced by maternal factors, with microbial transmission occurring through the vagina, gut, skin, and breast milk. Early-life stress (maternal stress, adversity) modulates intestinal permeability and microbial composition, promoting immune activation and sustained HPA engagement. In turn, cortisol and catecholamines reshape microbial niches, suggesting a bi-directional, feed-forward loop within the pediatric microbiota–gut–brain axis that may sensitize neurodevelopmental trajectories.

Gestational age affects composition, as preterm infants often exhibit dysbiotic profiles dominated by facultative anaerobes like Enterobacteriaceae, Enterococcus, and Staphylococcus [6]. Mode of delivery is a key determinant. Cesarean section (CS) is associated with altered microbiota, increased risks of immune dysregulation, infections, allergies, and inflammation. Intrapartum antibiotic use further disrupts colonization. Vaginal delivery (VD) allows vertical transmission of maternal vaginal and fecal microbiota, promoting stable colonization [7]. VD infants have higher levels of beneficial Bifidobacterium spp. and fewer potentially pathogenic genera like Enterococcus and Klebsiella. Proinflammatory genera, such as Klebsiella and Enterococcus, are more abundant in CS infants [8,9]. Notably, elevated Klebsiella levels can persist and a high Klebsiella/Bifidobacterium ratio in early life correlates with increased risk of pediatric allergic diseases [10].

### 3.2. Breastfeeding vs. Formula Feeding

Human milk, once thought sterile, contains commensal bacteria that shape early gut microbiota [11,12]. Its microbial composition may be altered by physiological stress and hormonal changes [13]. Rich in human milk oligosaccharides (HMOs), breast milk supports the growth of Bifidobacteria, fostering a microbiome dominated by this genus in early life [14]. To mimic this, some formulas are supplemented with probiotics, though not all strains are equally safe or effective, and results cannot be generalized across strains, even within species [15]. Despite Bifidobacteria supplementation, formula-fed infants still differ microbiologically from breastfed peers [16]. While bovine milk contains oligosaccharides structurally similar to HMOs, prebiotic supplementation may help develop a microbiota resembling that of breastfed infants, enhancing mucosal barriers and reducing pathogen translocation [17].

### 3.3. Dietary and Antibiotic Effects on the Gut Microbiota

Urbanization, reduced microbial exposure (e.g., through increased hygiene), and widespread antibiotic use have altered microbiota development. A critical window is the transition from breastfeeding to complementary feeding. The impact of breastfeeding post-weaning depends more on exclusive breastfeeding duration than on the timing of weaning. Early introduction of solids has been linked to shifts in microbiota composition and BMI [18], while dietary diversity, especially intake of protein and fiber-rich foods like meat, cheese, and rye bread, increases alpha diversity [19]. Children, the group most exposed to antibiotics, second only to analgesics, are particularly vulnerable to long-term effects, including allergies, asthma, obesity, autoimmune conditions, and neurodevelopmental disorders [20]. These effects are largely attributed to microbiota disruptions during its formative phase in the first 2–3 years. Children exhibit greater susceptibility to antibiotic-induced dysbiosis than adults, whose microbiota is more stable [21]. Certain antibiotics (e.g., penicillins, cephalosporins, carbapenems, macrolides, aminoglycosides) reduce beneficial taxa, whereas trimethoprim/sulfamethoxazole shows less impact [22]. Dysbiosis induces a pro-inflammatory state, though mechanisms remain unclear. Breastfeeding, and the use of prebiotics, probiotics, and postbiotics, may help mitigate antibiotic effects. ESPGHAN recommends specific probiotics—Saccharomyces boulardii and Lactobacillus rhamnosus GG—for preventing antibiotic-associated diarrhea [23,24].

**Figure 3:**
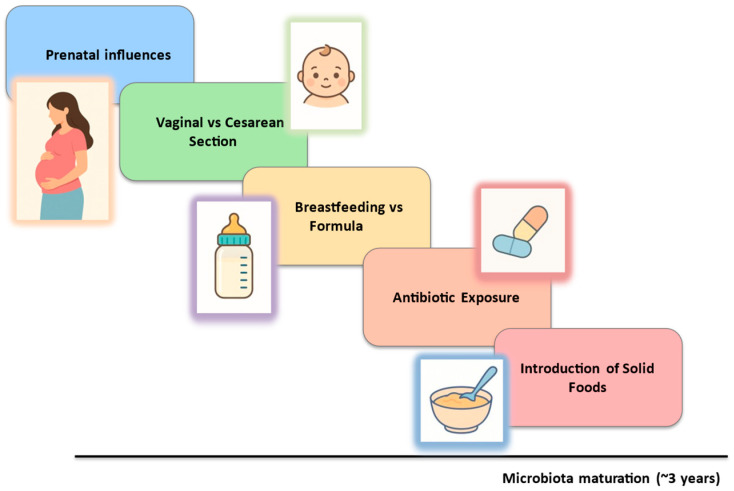
Early-life microbiota development timeline. Note. Graphical representation of key developmental windows shaping the gut microbiota from prenatal period to early childhood. Influences include maternal microbiome and in utero environment, mode of delivery, feeding modality, exposure to antibiotics, and introduction of solid foods, with microbiota composition approaching a more stable, adult-like configuration around three years of age.

A schematic overview of key early-life factors influencing microbiota development is shown in Figure 3.

## 4. The Microbiota and Brain Development

### 4.1. Influence on Neurobiological Development

Gut microbiota plays a key role in neurodevelopment. Germ-free (GF) mouse models lacking commensal bacteria show hyperactivity and reduced anxiety-like behaviors [25]. In humans, brain development begins in utero, involving neural progenitor proliferation, migration, differentiation, and integration into circuits, continuing postnatally through synaptogenesis, pruning, myelination, and adult neurogenesis [26]. By age five, the brain reaches over 85% of its adult volume, with near-complete myelination and established connectivity patterns [27]. These processes occur within sensitive periods (SPs) of heightened plasticity [28], whose timing and duration may be partially modulated by signals from the gut microbiome [29,30], suggesting new opportunities for early detection and intervention in atypical development.

### 4.2. Interaction with the Central Nervous System

Microbe-associated molecular patterns (MAMPs) from colonic bacteria can cross intestinal tight junctions, activate macrophages, and trigger TNF-α and IL-6 release. Microbial metabolites like SCFAs interact with immune cells (T cells, B cells, macrophages), inducing secretion of cytokines (TGF-β, IL-6, TNF-α) that reach the bloodstream and activate the vagus nerve, transmitting signals to the CNS. CD4^+^ T cells in the brain can activate microglia, promoting the expression of MHC-II, IL-10, and IL-2, and releasing TNF-α, which affects neuronal stem cell proliferation and microglial activation [31].

### 4.3. Role in Blood–Brain Barrier Development, Neuroinflammation, and Neurotransmitters

The gut microbiome is critical for blood–brain barrier (BBB) formation. GF mice show increased BBB permeability, which normalizes after colonization or SCFA administration [32]. Microglia, key immune cells in the CNS, regulate neuroinflammation, neurogenesis, and synaptic organization [33]. Their activation or inhibition influences both neurodevelopmental and neurodegenerative conditions [34]. In infants, Bacteroides species are prominent, metabolizing HMOs, mucin, and polysaccharides [35,36,37], and producing SCFAs, vitamin B6, folate, biotin, and lipoic acid. Bifidobacterium species support early colonization and immune development, producing lactate and acetate, which fuel colonocytes, stimulate serotonin (5-HT) release, and generate neuroactive compounds like GABA. Acetate also promotes Treg cell differentiation and strengthens gut barrier function [38,39,40].

SCFAs, particularly butyrate, propionate, and acetate, modulate the gut–brain axis by acting on microglia and astrocytes [41,42]. Microglia assist in synaptic pruning; astrocytes maintain BBB integrity and regulate metabolism. SCFAs enhance neurotransmitter release, synaptic plasticity, learning, and memory. Propionate specifically influences BBB gap junction gating [43,44,45].

## 5. Correlation Between Microbiota and Neuropsychiatric Disorders in Developmental Age

### 5.1. Gut Microbiota and Autism Spectrum Disorder (ASD)

An increasing amount of research has underscored a significant occurrence of gastrointestinal (GI) issues among people with Autism Spectrum Disorder (ASD), yet the exact mechanisms that connect these issues to the fundamental characteristics of ASD are still not fully understood.

Two main theories have been proposed to account for the origins of GI problems in this demographic [46]. The first, the inflammatory hypothesis, suggests that these symptoms could stem from chronic low-grade inflammation caused by dysbiosis of gut microbiota, a process that has been well-established in other GI disorders like inflammatory bowel disease and functional gastrointestinal disorders [47].

The second theory, the functional GI disorder hypothesis, indicates that these problems may be due to visceral hypersensitivity and changes in gut–brain communication, rather than any clear structural or metabolic abnormalities.

Evidence increasingly supports the idea that gut dysbiosis, gastrointestinal symptomatology, and the severity of ASD behaviors are closely interconnected. Notably, gastrointestinal complaints in children with ASD frequently occur without any identifiable anatomical or metabolic issues [48]. A meta-analysis conducted by McElhanon and colleagues (2014) revealed that children with ASD were significantly more likely than their neurotypical counterparts to experience a variety of gastrointestinal problems: they were about four times more likely to suffer from general gastrointestinal symptoms, three times more likely to present with constipation or diarrhea, and twice as likely to report abdominal pain. The connection between gastrointestinal disturbances and ASD goes beyond simple co-occurrence. Numerous studies have indicated that particular gastrointestinal symptoms-especially constipation and abdominal pain-are strongly linked to increased behavioral severity in ASD. These associations have been validated using standardized diagnostic and observational methods [49] and are often reflected in externalizing behavioral expressions, such as irritability or aggression [50]. Constipation, in particular, seems to be the most commonly reported gastrointestinal symptom among children with ASD [51] while abdominal discomfort has been directly associated with an increase in the severity of core ASD symptoms [52].

Furthermore, gastrointestinal symptoms in individuals with Autism Spectrum Disorder (ASD) are often linked to a range of additional comorbidities, such as issues with sleep, emotional regulation, and social interactions. Children with ASD who experience gastrointestinal problems are more likely to exhibit behavioral dysregulation, which may include self-injurious actions, aggression, oppositional behaviors, and challenges in expressive language and social reciprocity [53]. Research into the gut microbiota of those with ASD has shed light on the biological factors contributing to these gastrointestinal symptoms. A number of studies have reported significant changes in microbial composition that seem to align with particular gastrointestinal issues. For example, children with ASD who also have constipation have been found to have a higher relative abundance of certain bacterial groups, including Escherichia/Shigella and members of Clostridium cluster XVIII along with Fusobacteriales, Actinomycetaceae, Fusobacterium, Barnesiella, Coprobacter, Olsenella, and Allisonella [54,55]. In contrast, there is a notable decrease in beneficial bacteria such as Faecalibacterium prausnitzii, Bacteroides eggerthii, Bacteroides uniformis, Oscillospira plautii, and Clostridium clariafivum in these individuals [56], a consistent reduction in Lactobacillus species has been linked to chronic constipation in ASD, reflecting similar findings in non-ASD children experiencing comparable gastrointestinal issues [57,58].

In instances where ASD coincides with food allergies, the gut microbial composition seems to undergo further changes. Notably, there is frequently a higher prevalence of the phylum Proteobacteria, a category of bacteria that has been previously linked to immune-mediated and autoimmune disorders [59]. Furthermore, an increase in the Firmicutes/Bacteroidetes ratio has been observed in these individuals, a microbial characteristic typically associated with enhanced allergic immune reactions [59,60]. Certain microbial taxa have been associated with various GI phenotypes in ASD. For instance, both Clostridium aldenense and Oscillospira plautii have been found in children displaying general GI issues and constipation [61]. Additionally, Turicibacter sanguinis has surfaced as a particularly significant species, often found in higher levels in children with ASD and GI symptoms, and positively correlated with the severity of autistic traits [62].

Previous research reinforces these findings, indicating higher fecal concentrations of Clostridium histolyticum, a recognized toxin-producing bacterium, in children with ASD when compared to unrelated neurotypical controls. However, this distinction was not evident when comparing ASD children to their healthy siblings [63]. The ongoing presence of Clostridium species in ASD populations, regardless of overt gastrointestinal issues, highlights their probable significance in the gut–brain pathophysiology associated with the disorder.

### 5.2. Gut Microbiota and ADHD

Recent research has highlighted a significant connection between childhood ADHD and changes in gut microbiota. Longitudinal studies indicate that the composition of microbiota in early life could predict future neurodevelopmental outcomes. For example, Pärtty et al. (2015) showed that infants with higher levels of Bifidobacterium in their gut microbiota had a lower risk of developing ADHD later in childhood [64]. In a similar vein, Cassidy-Bushrow et al. (2023) performed a birth cohort study and discovered that infants who later developed ADHD had considerably fewer lactic acid bacteria, especially Lactobacillales and Enterococcaceae, in their microbiota at just six months old [65]. In addition to longitudinal studies, an increasing number of cross-sectional studies have investigated microbial variations in children already diagnosed with ADHD. Utilizing 16S rRNA sequencing, Aarts et al. (2017) found that children with ADHD exhibited a decrease in Firmicutes and an increase in Actinobacteria and Bifidobacterium when compared to healthy controls [66]. Similarly, Prehn-Kristensen et al. (2018) noted a significant reduction in alpha-diversity among ADHD patients, with marked differences in microbial composition, including a higher abundance of Bacteroidaceae, Neisseriaceae, and Neisseria spp., which were all proposed as potential microbial markers for ADHD [67].

Other researchers have pinpointed certain taxa that may be associated with ADHD symptoms. Jiang et al. noted a significant reduction in Faecalibacterium within the ADHD group, and this reduction was inversely related to the severity of symptoms as reported by parents [68]. Stevens et al. (2019) further validated the importance of microbial composition by connecting the abundance of Bifidobacterium to symptom scores in children diagnosed with ADHD [69]. In contrast, Wang et al. (2020) reported an increase in microbial diversity among ADHD patients, while also observing a decline in Bacteroides coprocola and a rise in Bacteroides ovatus, Sutterella stercoricanis, and Bacteroides uniformis [70]. In a larger sample, Szopińska-Tokov et al. (2020), building on Aarts’ research, identified seven genera that were significantly correlated with ADHD symptom scores [66,71]. Subsequently, Wang et al. (2022) found elevated levels of Agathobacter, Anaerostipes, and Lachnospiraceae UCG-010 in children with ADHD, along with decreased plasma TNF-α, which was negatively correlated with both microbial diversity and the severity of ADHD symptoms [72]. In the same year, Lee et al. (2022) linked Agathobacter to withdrawal and depressive symptoms, while Ruminococcus gnavus was associated with behavioral defiance [73]. Bundgaard-Nielsen et al. (2023) discovered increased levels of Eggerthella in children with ADHD, consistent with Aarts’ findings, and decreased levels of Colobacterium, Cholera, and Hodgkinella spp., which aligned with Prehn-Kristensen’s results [66,67,74].

Despite variations in findings across different studies due to methodological discrepancies and the diversity of populations, the persistent observations of modified gut microbiota profiles in ADHD emphasize the promise of microbiome research in revealing the pathophysiology of ADHD. Furthermore, the field has progressed beyond 16S rRNA methodologies; metagenomic techniques have enhanced the precision of microbial analyses. For instance, Wan et al. (2020) noted no significant change in alpha diversity but marked differences in microbiota composition, including reduced levels of Faecalibacterium and Veillonellaceae, alongside increased Odoribacter and Enterococcus [75]. Analyses at the species level indicated declines in Ruminococcus gnavus, Faecalibacterium prausnitzii, and Lachnospiraceae bacterium, while Paraprevotella xylaniphila, Odoribacter splanchnicus, Veillonella parvula, and Bacteroides caccae showed increases, corroborating findings by Jiang et al. [68,75]. Li et al. (2022) performed a comprehensive metagenomic analysis on 207 fecal samples, discovering heightened levels of Prevotella species (amnii, buccae, and copri) and Bifidobacterium species (breve and bifidum) in children diagnosed with ADHD, aligning with earlier observations by Aarts [76]. Moreover, the addition of Bacteroides ovatus was found to enhance spatial working memory and normalize EEG rhythms in animal models of ADHD [76]. Stiernborg et al. (2023) investigated the impact of neuroleptics and reported that children receiving drug treatment showed diminished microbiota functionality, altered beta-diversity, and a lower abundance of Bacteroides stercoris CL09T03C01, as well as genes related to vitamin B12 synthesis [77]. In studies conducted in 2023 and 2024, Wang et al. further underscored the rise in fungal taxa such as Ascomycetes and Candida in ADHD [78], along with increased levels of Anaerostipes hadrus, Lachnospira, and Phascolarctobacterium faecium, indicating their potential as microbial biomarkers [79].

### Possible Mechanisms Linking Gut Microbiota Dysbiosis and ADHD

The human gut contains a diverse ecosystem of microorganisms (bacteria, viruses, archaea, fungi, and protozoa) that play essential roles in physiological functions, such as vitamin production, immune system regulation, and defense against pathogens [80,81,82]. Notably, increasing evidence indicates that communication between the gut and brain occurs through various pathways: neural, neuroendocrine, and immune [83,84,85,86].

The autonomic nervous system (ANS) and the enteric nervous system (ENS) work together to regulate gut function. The vagus nerve, a crucial component of the ANS, influences emotions and stress through the hypothalamic–pituitary–adrenal (HPA) axis [87]. Research in animals has demonstrated that stimulating vagal afferents can alter reward-related behaviors, while vagotomy has been shown to reduce aggression in models of ADHD [88]. The microbiota may affect behavior through vagal signaling, as evidenced by anxiety-like behaviors triggered by pathogenic infections (Campylobacter jejuni, Citrobacter) [89]. Probiotic strains like Lactobacillus rhamnosus GG have been found to influence GABA receptor expression via the vagus nerve and alleviate anxiety symptoms [90].

The ENS, which interacts with the CNS, also plays a crucial role in maintaining the integrity of the intestinal barrier. Dysfunction in the ENS is associated with various gastrointestinal and neurological disorders, including ASD [91,92,93]. Gut microbes affect the maturation of the ENS through mechanisms that involve Toll-like receptors and the expression of 5-HT [94,95]. The disruption of microbiota due to antibiotics leads to a reduction in glial cells, changes in the structure of the ENS, and a decline in its functionality [96]. In germ-free (GF) mice, there is a notable decrease in ENS excitability, indicating that microbiota is vital for the development of the ENS [97]. The restoration of microbiota or the supplementation of short-chain fatty acids (SCFAs) enhances ENS function and increases glial cell counts [98,99]. These results suggest that dysfunction in the gut–brain axis, mediated by the ENS and ANS, may play a role in the pathophysiology of ADHD.

The symptoms of ADHD are closely linked to dysfunctions in critical neurotransmitter systems, such as dopamine (DA), norepinephrine (NA), GABA, and serotonin (5-HT) [100,101,102,103,104]. The gut microbiota has the ability to synthesize these neurotransmitters or their precursors. For example, altered dopamine signaling—particularly through D1 and D2 receptors—is associated with cognitive symptoms of ADHD [105], while reduced levels of 5-HT are connected to impulsivity and hyperactivity [106,107]. GABA, which is the primary inhibitory neurotransmitter, plays a role in regulating excitability and focus, and is often found to be deficient in individuals with ADHD [108]. SCFAs, which are products of microbial fermentation, affect CNS activity by modulating glucose metabolism and immune responses [109].

Notably, gut bacteria can produce catecholamines such as DA and NA [110]. Asano et al. found that GF mice had lower luminal DA and NA levels, which were restored after fecal transplantation from SPF animals [111]. Bacteria such as Prevotella and Bacteroides may affect dopamine.

### 5.3. Gut Microbiota and Bipolar Disorder

In recent years, the study of gut microbiota has gained prominence in the exploration of psychiatric disorders among children and adolescents, especially in cases like bipolar disorder (BD). This condition is marked by alternating mood episodes, ranging from depressive to (hypo)manic states. Pediatric BD has a lower occurrence rate of 1.8% compared to 3.1% in adults, and it frequently manifests with ultra-rapid and ultra-cycling mood changes, complicating the diagnostic process [112,113]. In addition to a significant genetic component (with heritability estimates between 70% and 90%), environmental factors can also trigger or worsen the disorder. There is growing evidence of a persistent inflammatory condition in individuals with BD, which is linked to irregularities in cytokines, immune markers, and T-cell activation [114]. These inflammatory reactions may be associated with alterations in gut microbiota. For example, higher levels of Escherichia coli and Bifidobacterium adolescentis are observed during manic phases, while Stercoris is more prevalent in depressive episodes [115]. Additional research has indicated lower levels of Faecalibacterium and Ruminococcaceae in BD patients, which negatively correlate with the duration of the illness and the severity of depressive symptoms [116,117]. These microbiota patterns are also connected to inflammation, metabolic indicators, and neurochemical disruptions. Probiotic treatments have shown potential benefits: Lactobacillus rhamnosus GG has been found to significantly lower rehospitalization rates, particularly in patients with elevated baseline inflammation [118] and other research has indicated cognitive enhancements following probiotic administration in euthymic individuals [119]. Nevertheless, anti-inflammatory therapies like infliximab did not seem to affect the microbiota [120].

### 5.4. Gut Microbiota and Major Depressive Disorder

Major depressive disorder (MDD) impacts around 2.6% of children and adolescents [121], with its prevalence rising during adolescence, especially among females [122]. Symptoms vary by age, often manifesting as irritability or aggression in younger children, which leads to underdiagnosis [123]. The disorder is multifaceted, involving biological, psychological, and environmental factors. Recently, research has concentrated on the connection between depression and gut microbiota. Numerous studies have identified significant compositional variations in the gut flora of depressed individuals compared to healthy controls [124,125], although findings regarding alpha diversity are inconsistent: some studies indicate increased diversity [125], while others show decreased diversity [126] and some report no difference at all [127]. Nevertheless, beta diversity, which reflects compositional dissimilarity between groups, has consistently demonstrated significant differences between depressed patients and healthy individuals [127,128]. These results imply a possible therapeutic role for dietary and microbiota-targeted interventions, such as probiotics or nutritional strategies, in treating and preventing depressive symptoms in youth [129,130].

### 5.5. Gut Microbiota and Anorexia Nervosa

Anorexia nervosa (AN), recognized as the most severe eating disorder, predominantly impacts adolescent females (up to 4%) and a smaller percentage of males (up to 0.3%) [131,132]. This condition is marked by a significantly low body weight resulting from restrictive behaviors that are not related to food availability or other medical issues [133] (World Health Organization, 2018). Besides genetic predisposition, psychosocial and interpersonal elements may initiate the disorder, while changes in neural circuits contribute to its persistence [134]. Given its primary focus on nutrition, AN significantly influences the gut microbiome. Numerous studies have indicated a decrease in alpha diversity among AN patients compared to healthy individuals, even during various stages of refeeding [135,136,137]. Methanobrevibacter smithii has often been identified in greater abundance in AN cases and shows a negative correlation with BMI. Other microbial changes include a reduction in Roseburia, Ruminococcus, and Clostridium, along with variations in short-chain fatty acids (SCFAs) such as butyrate and propionate, which are associated with anxiety and metabolic indicators [138]. Individuals with AN also exhibit lower levels of neurotransmitters and metabolites, including serotonin, dopamine, GABA, and acetate [139], although the precise connection to microbiota is still not fully understood. Variations in microbial composition have also been noted between restrictive and binge-purging types of AN, indicating that microbiota profiles may differ by subtype [139]. Importantly, alpha diversity appears to improve following short-term weight recovery, although beta diversity may still differ from that of healthy individuals [140]. These observations bolster the increasing interest in probiotic and microbiome-based treatments for adolescents suffering from AN [141].

### 5.6. Gut Microbiota and Social Anxiety

In a longitudinal birth cohort study that tracked children from one month old into their teenage years, researchers utilized the Dirichlet Multinomial Mixtures (DMM) method to analyze gut microbiota patterns during puberty. They identified four unique microbial clusters in children aged 12 to 14 years [142]. One of these clusters, named Puberty_3, stood out due to its notably low levels of Faecalibacterium, which was significantly linked to heightened social anxiety symptoms at age 14 [142]. This connection was further corroborated by a cross-sectional negative correlation between the relative abundance of Faecalibacterium and social anxiety scores, indicating a possible role for this taxon as a microbial marker for anxiety-related psychopathology in early adolescence [142]. Although Puberty_3 also contained higher levels of other genera such as Bifidobacterium, Akkermansia, Subdoligranulum, Christensenellaceae R-7 group, and Dialister, none of these exhibited a statistically significant cross-sectional relationship with social anxiety at age 14, underscoring the particular importance of Faecalibacterium in this scenario [142].

Previous research has indicated lower levels of Faecalibacterium in individuals with generalized anxiety disorder (GAD), as well as in various psychiatric groups, including those suffering from MDD, bipolar disorder, and autism spectrum disorder (ASD) [143,144,145,146]. Specifically, a decrease in Faecalibacterium abundance has been associated with the intensity and length of social exclusion experiences among young adults, implying that this genus might have a more extensive role in influencing social cognition and stress-related behaviors [145]. From a mechanistic standpoint, Faecalibacterium prausnitzii—the most prevalent and well-studied species in this genus—is recognized for its ability to produce butyrate, a short-chain fatty acid known for its strong anti-inflammatory effects [147,148]. In addition to its immunomodulatory functions, butyrate also affects gut–brain communication by regulating gut hormone levels, which are involved in appetite control and mood, potentially influencing behavioral and emotional regulation during critical developmental phases such as puberty [149,150].

The strength of this study is attributed to its longitudinal design and the utilization of a well-defined community sample, enabling the authors to track microbiota trajectories from early life to adolescence and connect them to behavioral outcomes within a developmental context [142]. Additionally, by grouping children based on microbiota composition instead of isolated taxa, the authors clarified the intricate relationships between microbial diversity and behavior, while accounting for various confounding factors such as diet, sleep, and alcohol consumption [142]. Importantly, although three out of the four pubertal clusters were similar to previously identified adult enterotypes, the Faecalibacterium-depleted Puberty_3 cluster seemed less stable and may indicate a less mature or more transitional state of microbiota [142]. These results emphasize the potential role of Faecalibacterium as a significant microbial marker of social anxiety in young individuals and stress the relevance of gut microbiota composition as a potential biomarker and modifiable element in the prevention and treatment of anxiety disorders. Nevertheless, since this is an observational study, causal relationships cannot be established. Future investigations should focus on validating these results through mechanistic studies, including metagenomic and metabolomic analyses, and ideally through interventional trials [142,151,152].

Given the heterogeneity of findings, Table 2 summarizes the most consistent microbiota alterations described in children and adolescents with neuropsychiatric disorders, highlighting disorder-specific microbial signatures and their potential clinical implications.

## 6. Possible Interventions and Therapeutic Implications

### 6.1. Probiotics/Psychobiotics and Mental Health

The concept of psychobiotics refers to probiotics, prebiotics, or synbiotics capable of influencing gut neurotransmission and, consequently, mental health [153]. Interest in their psychiatric applications has grown considerably in recent years, even though current findings remain inconsistent [154]. Despite the absence of robust regulatory frameworks or systematic cost–benefit evaluations, psychobiotics are widely marketed as over-the-counter supplements, with particular uptake in low-resource settings where they are perceived as accessible adjunctive tools [155].

Clinical studies have examined their role in anxiety, depression, and other psychiatric conditions, though results have been heterogeneous. In anxiety disorders, probiotics containing strains such as Lactobacillus and Bifidobacterium have been associated with improvements in general well-being and reductions in negative symptoms [156]. Additional trials have shown modulation of immune and endocrine responses under stress [157,158] and even enhancement of cognitive flexibility [159]. Evidence from patients with comorbid medical conditions suggests further potential for reducing anxiety symptoms [160,161], and preliminary data point to possible gene–environment interactions, such as IL-1β polymorphisms that may modulate individual responses [162].

In depression, the evidence is similarly mixed. Early studies indicated that probiotics could attenuate depressive symptoms and decrease inflammatory markers [163,164], yet later randomized controlled trials reported inconsistent improvements in mood, anxiety, and stress [165]. Nevertheless, adjunctive approaches, such as combining probiotics or synbiotics with conventional antidepressants like fluoxetine, have demonstrated promising results, including improved depressive scores and increased microbial diversity [166,167]. Mechanistic evidence suggests that these benefits may involve modulation of neurotrophic factors such as BDNF [168,169], structural changes in fronto-limbic brain networks [170], and reduced inflammatory signaling [171]. Overall, psychobiotics appear safe and well tolerated, but systematic reviews consistently emphasize the need for larger, strain-specific studies with longer follow-up before clinical recommendations can be made [172,173,174,175].

Probiotics were systematically evaluated in relation to depression across randomized controlled trials [176]. The analysis confirmed a modest but significant reduction in depression scores compared to placebo, with potential benefits observed in both depressed and non-depressed populations. Preclinical findings complement these results: disruption of the microbiome induces depressive- and anxiety-like behaviors in rodents [177], while Bifidobacterium infantis has been shown to reverse stress-induced immune and behavioral deficits [178] and even outperform escitalopram in certain models [179]. Human RCTs provide converging evidence, with Lactobacillus helveticus R0052 and Bifidobacterium longum reducing stress and depressive symptoms in healthy volunteers [180], multispecies probiotics improving biomarkers in workers [181], and probiotic yogurt enhancing mood in individuals with poor baseline affect [182]. Importantly, Akkasheh et al. [183] reported a significant clinical improvement in patients with MDD, supporting the potential for probiotics as adjunctive treatments, though Huang et al. (2016) emphasized that strain heterogeneity, small sample sizes, and geographic variability remain major limitations [176].

Additional insight comes from a systematic review by Renata S. D. Barbosa (2020) [184], which restricted its scope to studies involving patients with formal psychiatric diagnoses. In schizophrenia, probiotics did not significantly improve core symptoms, though benefits were evident in patients with gastrointestinal comorbidities or Candida albicans seropositivity [185,186]. In BD, probiotics reduced rehospitalization rates and shortened hospital stays during acute mania, especially in patients with higher systemic inflammation [187]. For major depressive disorder, all three included trials reported clinical improvements with probiotics, but not with prebiotics or placebo, with one study showing modulation of serotonin metabolism via a reduction in the kynurenine/tryptophan ratio [183,188]. In autism spectrum disorder, probiotic and prebiotic interventions, particularly when combined with dietary modifications, showed beneficial effects on gastrointestinal symptoms as well as anxiety and irritability [189,190,191,192]. Long-term data also suggest preventive potential: a 13-year prospective study found that probiotic administration from pregnancy through infancy reduced the risk of ADHD and ASD, even though the precise microbiota-related mechanisms remain unclear [64].

Mechanistically, psychobiotics are thought to act through several interconnected pathways. These include modulation of immune signaling and cytokine balance, reduction in intestinal permeability with a consequent decrease in systemic inflammation, alterations in tryptophan metabolism affecting serotonin and kynurenine pathways, and regulation of microbial metabolites such as valeric, propionic, and butyric acids [193,194]. Importantly, safety data across psychiatric trials remain reassuring, with only mild gastrointestinal adverse events occasionally reported, and rarely leading to treatment discontinuation [185,189].

Taken together, psychobiotics represent a promising but still experimental frontier in mental health care. Evidence supports their adjunctive role in depression, bipolar disorder, and possibly other psychiatric conditions, yet inconsistencies across trials and methodological limitations preclude firm conclusions. Future research must therefore focus on large, strain-specific randomized controlled trials, mechanistic exploration, and long-term safety and cost–benefit evaluations before psychobiotics can be integrated into routine psychiatric practice.

### 6.2. Diet and Nutritional Interventions

Diet is one of the most influential environmental factors shaping the human gut microbiota, affecting both its composition and metabolic functions [195,196]. Dietary components dynamically interact with microbial ecology and host physiology, modulating microbial diversity, abundance of specific taxa, and production of bioactive metabolites that influence systemic and brain health [197,198]. Conversely, unhealthy dietary patterns can disrupt these interactions and have been consistently associated with increased metabolic and mental health risks.

Western-style diets, typically high in refined carbohydrates, saturated fats, sugar, and processed foods but poor in fiber and polyphenols, reduce microbial diversity, favor the expansion of pathobionts, and diminish commensal species [199,200]. These alterations are accompanied by an unfavorable metabolic profile, including reduced production of short-chain fatty acids (SCFAs) such as butyrate, propionate, and acetate, which regulate immune and neuroendocrine pathways [201,202,203]. In contrast, diets rich in fermentable fibers, whole grains, fruits, and vegetables exert prebiotic effects that enhance beneficial microbes such as Bifidobacterium and Lactobacillus spp., improve intestinal barrier integrity, and support anti-inflammatory signaling cascades [204]. The concept of “prebiotics” has recently been reframed as “microbiota-accessible carbohydrates” (MACs), underlining the crucial role of fermentable fibers in sustaining microbial metabolism [205]. Low-fiber diets reduce microbial diversity, erode the mucus barrier, and increase infection susceptibility [206,207,208]. Notably, different fibers exert distinct effects: for example, inulin may exacerbate colitis, whereas pectin appears protective [209].

Other dietary macronutrients also shape the microbiome in specific ways. High-fat diets reduce diversity and increase the Firmicutes/Bacteroidetes ratio, promoting pathobionts such as Bilophila wadsworthia, particularly with milk-fat consumption [210,211,212]. Conversely, omega-3 polyunsaturated fatty acids (PUFAs) can enrich beneficial taxa like Bifidobacterium and Roseburia [213]. Protein intake likewise modulates microbial composition and metabolite production: excessive protein fermentation generates hydrogen sulfide and ammonia, compounds linked to inflammation and epithelial damage [214,215]. Protein source is a key determinant: while animal proteins increase IBD risk [214,216,217] soy protein may enhance microbial diversity in Western diet models [218]. Moreover, tryptophan metabolism by gut microbes produces AhR ligands that stimulate IL-22 release, reinforcing mucosal integrity and exerting anti-inflammatory effects [219]. Other dietary components such as polyphenols and selenium promote beneficial microbial shifts [220,221], while high sugar intake and food additives like emulsifiers (CMC, P80) can disrupt the mucus barrier, increase Proteobacteria and Akkermansia muciniphila, and exacerbate colitis in susceptible hosts [222].

These findings have given rise to a growing interest in complex dietary patterns rather than isolated nutrients. The Mediterranean diet, rich in fiber, polyphenols, omega-3 fatty acids, and unsaturated fats, has been associated with increased microbial diversity, enrichment of beneficial taxa such as Faecalibacterium prausnitzii, Roseburia, and Bifidobacterium spp., and decreased pro-inflammatory bacteria [223,224]. Such microbiota-related changes have been linked not only to metabolic and cardiovascular benefits but also to improved mental health outcomes, including a lower incidence of depression.

The role of diet in shaping the microbiome is particularly evident in neurodevelopmental conditions such as autism spectrum disorder (ASD), where atypical eating behaviors are common. Children with ASD often display restrictive and selective eating patterns, influenced by sensory sensitivities and behavioral rituals, which lead to reduced intake of fruits, vegetables, and fibers, alongside increased consumption of processed, high-calorie foods [225,226]. This results in both overweight and undernutrition, as well as frequent micronutrient deficiencies (calcium, vitamin D, iron, zinc, vitamin B12, folate) with consequences for growth, bone health, and—in rare cases—clinical deficiencies such as scurvy [227,228]. These nutritional imbalances have been associated with reduced microbial diversity and altered SCFA production, potentially linking diet, gastrointestinal symptoms, and microbiota-related mechanisms in ASD [229,230].

Several dietary interventions have been tested in this population, though with heterogeneous and often inconclusive results. The gluten-free/casein-free diet (GF/CFD) remains the most common, with some studies reporting behavioral and gastrointestinal improvements, while others emphasize risks of reduced fiber intake and worsening gut health [231,232]. The ketogenic diet (KD), initially developed for refractory epilepsy, has shown preliminary benefits on seizures and behavior in ASD, but evidence is still limited and side effects significant [227,233]. Other approaches such as the specific carbohydrate diet (SCD) have only anecdotal support [234]. By contrast, the Mediterranean diet has not been systematically tested in ASD, but its beneficial microbiota-related effects observed in other psychiatric and neurodevelopmental conditions, such as ADHD, make it a promising avenue [235,236].

Altogether, diet emerges as a critical modulator of gut microbiota with downstream effects on systemic and brain health. While broad dietary principles such as increasing fiber and polyphenol intake are generally beneficial, the effects of specific interventions remain highly individualized, reflecting baseline microbiota composition and host factors [237,238]. Neurodevelopmental disorders like ASD illustrate how altered dietary patterns, nutritional deficiencies, and microbiota dysbiosis can interact, offering a window into the potential of targeted nutritional strategies within the broader microbiota–gut–brain axis.

**Figure 4:**
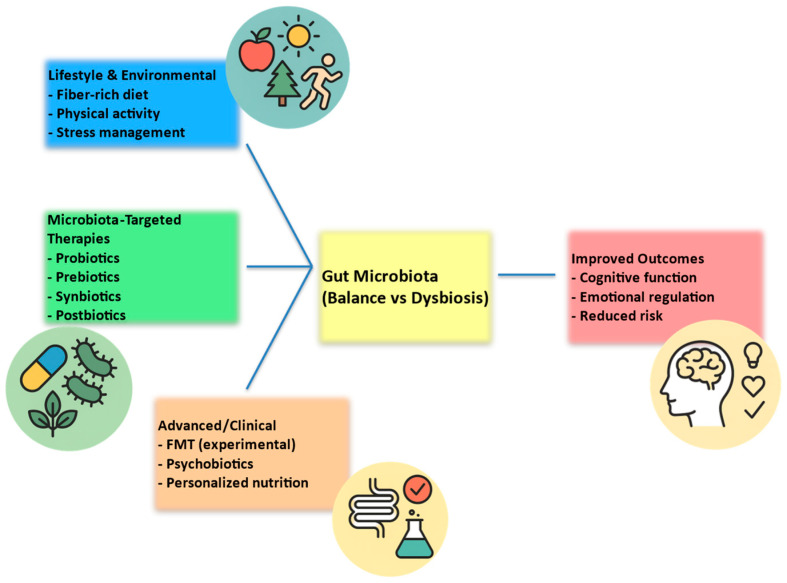
Potential interventions targeting the pediatric microbiota–gut–brain axis. Abbreviations: FMT = Fecal Microbiota Transplantation.

Figure 4 summarizes possible strategies, including lifestyle and dietary interventions, microbiota-targeted therapies, and advanced approaches such as psychobiotics and fecal microbiota transplantation.

## 7. Conclusions and Future Directions

Recent advances in microbiome research have highlighted a strong association between gut microbiota composition and the development of various neuropsychiatric disorders during childhood and adolescence. This review addresses a key gap by providing a pediatric-centric synthesis that integrates early-life determinants, mechanistic pathways of the microbiota–gut–brain axis, and disorder-specific evidence across ASD, ADHD, bipolar disorder, MDD, and anxiety.

Conditions such as ASD, ADHD, bipolar disorder, MDD, anorexia nervosa, and social anxiety have all been linked to specific alterations in the gut microbiota. These alterations often include a reduction in beneficial bacteria like Faecalibacterium prausnitzii and Bifidobacterium, as well as an increase in potentially harmful or pro-inflammatory microbes such as Clostridium and Escherichia. Importantly, these microbial imbalances are frequently associated with the severity of psychiatric symptoms, gastrointestinal comorbidities, and behavioral disturbances, suggesting that the gut–brain axis may play a key role in the pathophysiology of these conditions. Beyond bacteria, the pediatric gut virome (notably bacteriophages) and mycobiome increasingly appear to shape community dynamics, mucosal immunity, and metabolite availability. Although pediatric data remain sparse, integrating these axes is essential for a comprehensive microbiota–gut–brain axis model.

Despite the growing body of evidence, the exact nature of these relationships remains unclear. Most current studies are correlational, and it is not yet possible to determine whether gut dysbiosis is a cause, consequence, or merely a co-occurring feature of neuropsychiatric disorders. Additionally, inconsistencies across findings, often due to differences in study design, population characteristics, and microbial analysis methods, make it difficult to draw definitive conclusions. As this is a narrative review, no formal risk-of-bias assessment or quantitative synthesis was performed. The included studies vary in methodology, population characteristics, sequencing platforms, and taxonomic resolution, which introduces heterogeneity and limits causal inference. Therefore, associations between gut microbiota composition and neuropsychiatric outcomes should be interpreted carefully, and further mechanistic and longitudinal clinical studies are needed. Evidence remains predominantly associative, with limited causal inference. Heterogeneity arises from sequencing strategies and bioinformatic pipelines; sample handling and batch effects; confounding (diet/medications/genetics/geography); phenotype definition; and translation gaps from animal to human pediatric cohorts.

Nevertheless, early interventional research points to the potential of psychobiotics and dietary modifications as supportive strategies in managing certain psychiatric symptoms. Probiotic and prebiotic treatments have shown encouraging results in reducing anxiety, depression, and gastrointestinal discomfort, particularly in individuals with ASD, ADHD, and bipolar disorder. Looking ahead, future research must focus on clarifying these associations through well-designed, large-scale longitudinal and mechanistic studies. High-resolution metagenomic and metabolomic approaches could provide deeper insights into the specific microbial pathways involved in brain–gut communication. Moreover, understanding individual variability in microbiota composition and host factors will be essential for developing more targeted and effective therapeutic interventions. While microbiota-based treatments offer a promising and largely safe avenue, their integration into routine psychiatric care will depend on stronger clinical evidence regarding their long-term efficacy, safety, and cost-effectiveness.

## Figures and Tables

**Table 1:** Major factors influencing gut microbiota development in early life.

Factor	Characteristics	Impact on the Microbiota
Mode of delivery	Vaginal birth vs. cesarean section	Vaginal birth → higher microbial diversity; Cesarean section → reduced exposure to maternal microbes
Feeding	Breastfeeding vs. formula feeding	Breast milk → promotes Bifidobacteria dominance; Formula → more heterogeneous composition
Antibiotics	Early-life exposure	Reduced diversity, risk of persistent dysbiosis
Introduction of solids	Timing and diversity of complementary feeding	Increased microbial diversity, progressive maturation of the microbiota
Environment	Rural vs. urban settings	Rural exposure → richer microbial diversity; Urban/sterile environments → lower diversity

**Table 2:** Alterations of gut microbiota associated with major pediatric neuropsychiatric disorders.

Disorder	Microbiota Alterations	Key Findings/Associations
Autism Spectrum Disorder (ASD)	↑ Clostridium, Escherichia/Shigella; ↓ Faecalibacterium, Lactobacillus	Reduced microbial diversity; GI symptoms frequently correlated with severity of ASD behaviors
Attention-Deficit/Hyperactivity Disorder (ADHD)	↓ Faecalibacterium, Lactobacillales; ↑ Actinobacteria, Bacteroidaceae	Altered short-chain fatty acids; links with cognitive and behavioral regulation
Bipolar Disorder (BD)	↑ Escherichia coli, Bifidobacterium adolescentis; ↓ Faecalibacterium	Pro-inflammatory microbiota signature; potential role in mood instability
Major Depressive Disorder (MDD)	Reduced beta diversity; variable taxa changes	Impaired serotonin pathway metabolites; associated with altered gut–brain signaling
Anorexia Nervosa (AN)	↓ Roseburia, Ruminococcus; ↑ Methanobrevibacter smithii	Reduced SCFAs production; microbial shifts may contribute to weight regulation difficulties
Social Anxiety Disorder	↓ Faecalibacterium	Lower abundance linked with increased anxiety and altered stress response

Abbreviations: GI = Gastrointestinal.; SCFAs = Short-chain fatty acids. Arrows indicate directional changes in microbial relative abundance: ↑ denotes an increase, while ↓ denotes a decrease.

## Data Availability

No new data were created.

## Abbreviations

The following abbreviations are used in this manuscript:

SCFAs	short-chain fatty acids
HPA	hypothalamic–pituitary–adrenal
ANS	autonomic nervous system
ENS	enteric nervous system
BBB	blood–brain barrier
ASD	autism spectrum disorder
ADHD	attention-deficit/hyperactivity disorder
MDD	major depressive disorder
BD	bipolar disorder
FMT	fecal microbiota transplantation
MAMPs	microbe-associated molecular patterns
